# Cardiotoxicity associated with immune checkpoint inhibitors: Current status and future challenges

**DOI:** 10.3389/fphar.2022.962596

**Published:** 2022-08-30

**Authors:** Lu Gan, Demin Liu, Yanan Ma, Xuening Chen, Aihui Dai, Sihan Zhao, Xiaoxue Jin, Guoqiang Gu

**Affiliations:** ^1^ Research Laboratory of Emergency Medicine, Emergency Department, West China Hospital, Sichuan University, Chengdu, China; ^2^ Cardiology Department, Second Hospital of Hebei Medical University, Shijiazhuang, Hebei, China

**Keywords:** cardiotoxicity, immune checkpoint inhibitors, oncotherapy, tumor immunotherapy, myocarditis

## Abstract

Immune checkpoint inhibitors (ICIs) are the most notable breakthrough in tumor treatment. ICIs has been widely used in tumor patients, but its wide range of immune-related adverse events (irAEs) should not be ignored. irAEs can be involved in any organ system, including immune-related cardiotoxicity. Although the cardiotoxicity induced by immune checkpoint inhibitors is rare, it is extremely lethal and has attracted increasing attention. PD-1 and PD-L1 are expressed in human cardiomyocytes, so the application of PD-1/PDL-1 inhibitors can cause many adverse reactions to the cardiovascular system. This review summarizes the latest epidemiological evidence on the cardiovascular toxicity of programmed cell death protein-1(PD-1)/programmed cell death ligand-1(PD-L1) inhibitors and the clinical manifestations, as well as the potential pathological mechanisms. These updates may provide a novel perspective for monitoring early toxicity and establishing appropriate treatment for patients with ICI-related cardiotoxicity.

## Introduction

Tumor immunotherapy is an important clinical strategy for the treatment of various solid and hematological malignancies (Bhullar et al., 2018). Immune checkpoint inhibitors (ICIs) are the most notable breakthrough in tumor treatment. They significantly improve clinical efficacy and extend the overall survival time of cancer patients (Gong et al., 2018). ICI therapy achieves unprecedented durable antitumor responses, especially in advanced malignancies such as metastatic melanoma (Dummer et al., 2020), non-small cell lung cancer (Leonetti et al., 2019), renal cell carcinoma (Atkins et al., 2017), and refractory Hodgkin’s lymphoma (Haslam and Prasad, 2019).

The therapeutic mechanism of ICIs is based on targeting immunosuppressive checkpoints, including cytotoxic T lymphocyte-associated protein-4 (CTLA-4), programmed cell death protein-1 (PD-1), and PD-1 ligand (PD-L1), which prevents the immune escape response of tumor cells through reactivating inoperative cellular immunity mediated by these molecules (Ribas and Wolchok, 2018). Currently, numerous ICIs, such as anti-PD-1 (nivolumab or pembrolizumab), anti-PD-L1 (atezolizumab, avelumab, or durvalumab), and anti-CTLA-4 (ipilimumab or tremelimumab) antibodies, can activate the anti-tumor immune response and inhibit the growth of tumor cells (Weyand et al., 2018). Furthermore, the ICI combination therapy can further activate the function of T cells and produce a synergistic anti-tumor effect compared to that of single-drug therapies (Motzer et al., 2018; Wang et al., 2018).

While activating T cell anti-tumor effects, ICIs can produce a series of autoimmune toxicities due to the activation of more responsive T cells, resulting in the occurrence of immune-related adverse events (irAEs) (Postow et al., 2018). Approximately 60%–80% of patients treated with ICIs may experience irAEs, and 20% of them can experience high-grade irAEs (> grade 3; grade set forth by the Common Terminology Criteria for Adverse Events) (Khunger et al., 2020). Among patients with cancer treated with ipilimumab and nivolumab, more than 90% had at least one irAE, and approximately 50% had serious irAEs (Koelzer et al., 2016).

In tumor immunotherapy, ICIs can lead to the development of immune-related cardiotoxicity. Although ICI-related cardiotoxicity is rare, the mortality rate is extremely high (Hu et al., 2019). The fatality rate of ICI-associated myocarditis amounts to 35%–50% (Mahmood et al., 2018; Mir et al., 2018). In addition, patients treated with ICIs have a four-fold increased risk of major adverse cardiac events, including cardiovascular mortality and arrest, and cardiogenic shock (Moslehi et al., 2018). It is worth noting that patients treated with CTLA-4 blockades are more likely to develop cardiologic irAEs than patients treated with PD-1 inhibitors. Furthermore, patients treated with a combination of ICIs seemed to have a higher incidence of cardiotoxicity and mortality than those treated with a single ICI (Salem et al., 2018; Tawbi et al., 2018). The purpose of this review is to summarize the latest epidemiological evidence of cardiovascular toxic effects of PD-1/PDL-1 inhibitors as well as the clinical presentation and possible underlying mechanisms, in order to provide new strategies for clinical prevention and treatment and to minimize the incidence of fatal cardiac events associated with ICI treatments.

## Epidemiology

Reports of cardiotoxicity in ICI clinical trials are rare. The most common manifestation of cardiotoxicity is myocarditis (Supplemenatry Table S1). A retrospective study of data from eight clinical centers found that the prevalence of myocarditis was 1.14% (Mahmood et al., 2018). In a phase II trial of pembrolizumab (anti-PD-1) for the treatment of thymic epithelial tumors, 34% of patients had grade 3 or higher irAEs, of which 9% had myocarditis and 6% had myasthenia gravis (Cho et al., 2019). Moreover, according to the data from the International Database VigiBase of the World Health Organization Individual Case Safety Report, the proportion of myocarditis cases in patients treated with a combination of ICIs (1.33%) was higher than those treated with monotherapy (0.31%) (Salem et al., 2018). The median onset time of myocarditis was 17–34 days, but most cases were discovered within the first 3 months. More fulminant cases usually occurred after the first few ICI treatments, and more than 70% of cases occurred within the first 6 weeks of treatment (Waliany et al., 2021). In addition, secondary myocarditis due to treatment with a combination of ICIs had a higher mortality rate (67%) compared to the mortality rate of monotherapy (36%), which indicates that the myocarditis phenotype of combined ICIs is more severe (Moslehi et al., 2018).

A meta-analysis of 22 clinical trials of ICI treatment for patients with lung cancer reported that the incidence of ICI-related myocardial infarction was 1% (Khunger et al., 2020). Rapid progression of atherosclerosis has been observed in patients with giant cell tumors of the bone (Kwan et al., 2019). ICIs can cause tachyarrhythmias, including atrial fibrillation, supraventricular tachycardia, and ventricular tachycardia, and also brady arrhythmias, including partial or complete atrioventricular block (Agrawal et al., 2019). In patients receiving ICI combination therapy, QTd is prolonged, potentially indicating increased susceptibility to ventricular arrhythmia (Pohl et al., 2020).

## Clinical presentation of ICIs-related cardiotoxic effects

**FIGURE 1:**
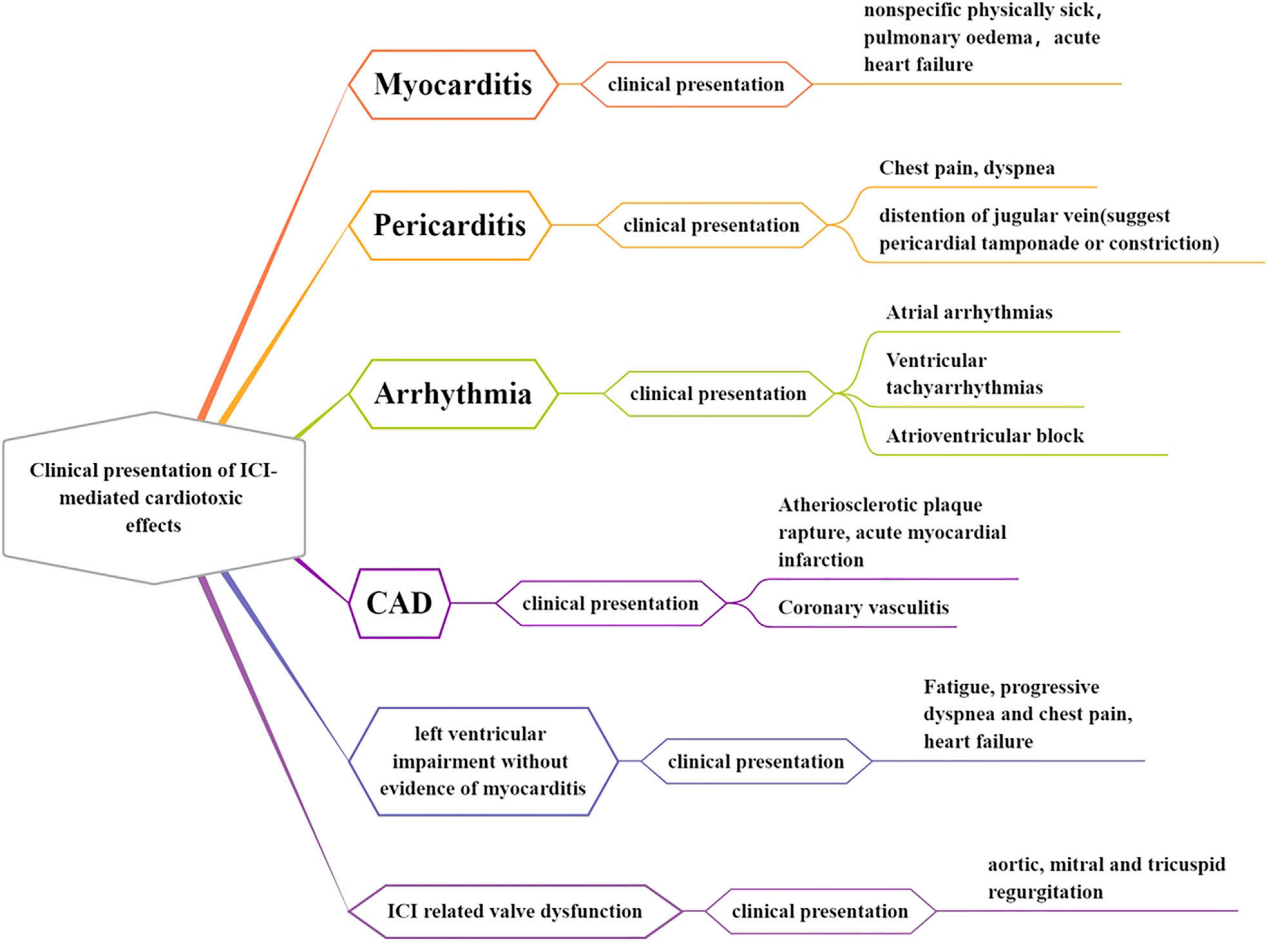
Clinical presentation of ICI-mediated cardiotoxic effects. The ICI-associated clinical trial and reports of current practice suggest that the manifestations of ICI-related cardiotoxicity are diverse, including myocarditis, pericardial disease, arrhythmia, myocardial infarction, and left ventricular (LV) dysfunction without evidence of myocarditis, such as Tako-Tsubo cardiomyopathy (TTC).

At present, there are no standard diagnostic criteria for the clinical diagnosis of ICI-related cardiotoxicity. A diagnosis of exclusion is generally based on clinical manifestations and auxiliary examination results. The results of the ICI clinical trial and reports of current practice suggest that the manifestations of ICI-related cardiotoxicity are diverse, including myocarditis, pericardial disease, arrhythmia, myocardial infarction, and left ventricular (LV) dysfunction without evidence of myocarditis, such as Tako-Tsubo cardiomyopathy (TTC) (Lyon et al., 2018) (Figure 1). Some of these complications can develop and progress rapidly within a few days or weeks after ICI treatment. However, several complications, such as chronic diseases, can increase the risk of cardiovascular disease-associated morbidity and mortality in the long term (Han et al., 2020).

ICI-related myocarditis is the leading early ICI-related cardiovascular adverse event, and it can be acute and fulminant (Dolladille et al., 2020). According to the IRAE classification system proposed by the American Society of Clinical Oncology (ASCO) clinical practice guidelines, the clinical manifestations of ICI-related myocarditis can range from elevated cardiac biomarkers to severe cardiac decompensation with multiple organ failure (Palaskas et al., 2020). ICI-related myocarditis is characterized by elevated levels of serum cardiac biomarkers (troponin T/I), brain natriuretic peptide (BNP), or NT-proBNP (N-terminal pro-BNP), new LV impairment, and the presence of active myocardial inflammation as revealed by cardiac MRI or endomyocardial biopsy (Chen et al., 2020). In addition, approximately 89% of patients with ICI-related myocarditis develop new-onset ECG changes (Mahmood et al., 2018). Additionally, ICI-related myocarditis can occur with cardiogenic shock, multiorgan failure, ventricular arrhythmias, and even death in severe cases (Chen et al., 2020). Therefore, it is of great clinical value to explore the pathogenesis of ICI-associated myocarditis.

ICI treatment can be associated with pericardial diseases, including pericarditis, pericardial effusion, and pericardial tamponade. ICI-related pericarditis can occur in isolation or alongside myocarditis (Lyon et al., 2018). Pericarditis is characterized by the presence of typical chest pain and dyspnea and can rapidly progress to respiratory failure (Chen et al., 2020). In terms of symptoms, patients may present with signs of pericardial friction, tamponade, or coarctation (Ala et al., 2019). ECG changes in such patients include PR depression, widespread saddle-shaped ST elevation, low QRS voltage, and T-wave inversion (Chen et al., 2020). Troponin elevation is seen in patients with pericardial disease accompanied by myocarditis, and is associated with a poor prognosis (Mahmood et al., 2018). ICI therapy can also result in cardiac conduction disease, including atrial fibrillation, ventricular arrhythmias (ventricular tachycardia or VF), and atrioventricular block (Lyon et al., 2018). Supraventricular arrhythmias have the highest incidence (Guo et al., 2020). Patients with ICI-related conduction disease can present with a prolonged PR interval, bundle branch block, and in severe cases, complete AV block, which may lead to cardiac arrest (Chen et al., 2020). In addition, ICI therapy can also lead to LV functional impairment without myocardial inflammation, manifesting only as a functional impairment. There are various forms of ICI-related impairment of LV function, which can occur concomitantly with dilated cardiomyopathy, such as TTC (stress-induced cardiomyopathy) (Lyon et al., 2018).

## Anti-tumor mechanism of ICIs

**FIGURE 2:**
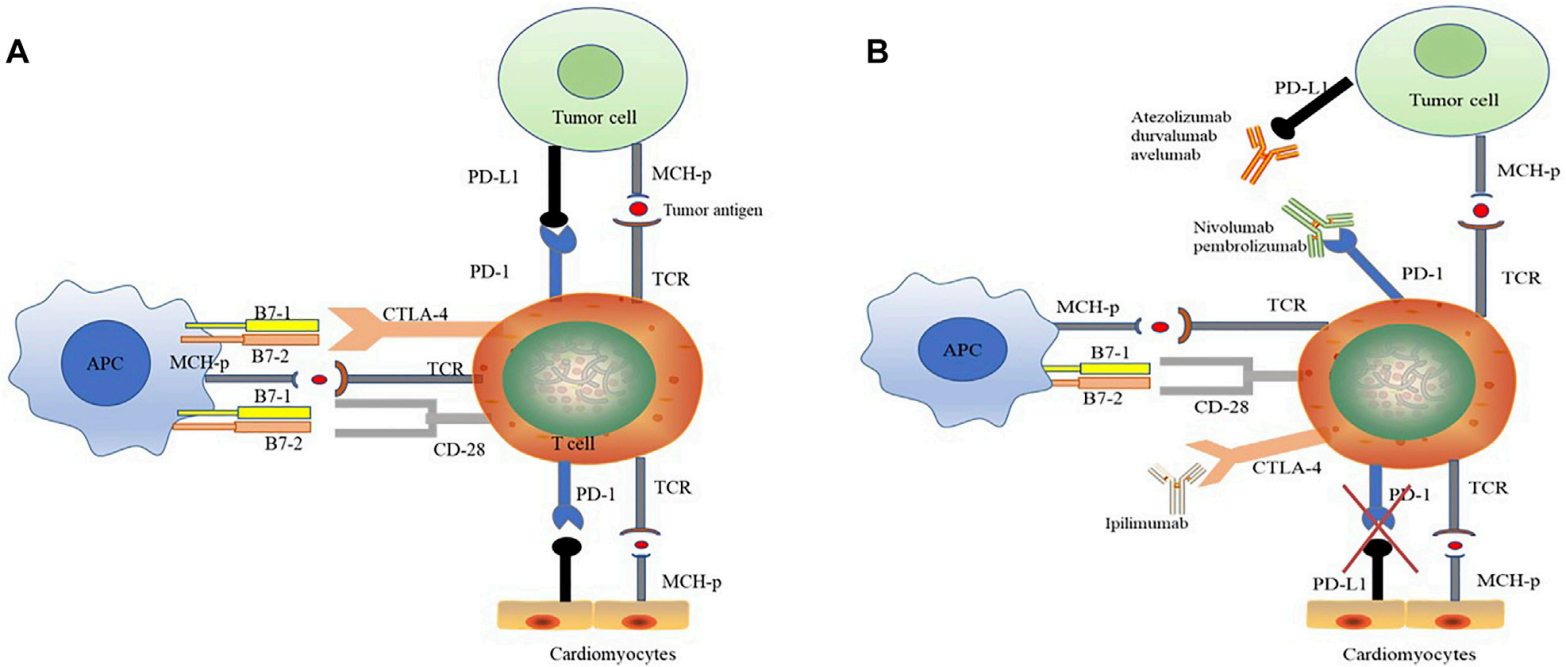
The anti-tumor effect of ICI therapy is associated with potential cardiotoxicity. **(A)** “PD-1–PD-L1 axis” induced immunosuppression. PD-1 is a member of the CD28 superfamily. PD-1 inhibits the activation of T cell receptor-proximal kinases and prevents the formation of stable T cell-APC contacts, thereby affects signal transduction and cell-effective functions mediated by T cell antigen receptors. PD-1 ligands (PD-L1 and PD-L2) are highly expressed in tumor cells, and the PD-1 expression of infiltrating T cells is upregulated under tumor microenvironment. PD-1 and PD-L1 combination suppresses T cell activation and disables the anti-tumor immune response. Meantime, PD-L1 is also expressed in cardiomyocytes, and the binding of PD-1 to myocardial PD-L1 suppresses the own immune response and maintains the homeostasis in heart. **(B)** ICIs have anti-tumor effects and potential cardiotoxicity. ICIs are neutralizing antibodies that completely bind to immunosuppressive checkpoints and thus exerts anti-tumor by activating immune response. PD-1 antibody (Atezolizumab, durvalumab and avelumab) and PD-L1 antibody (Nivolumab and pembrolizumab) respectively binding to PD-1 and PD-L1, breaking “PD-1–PD-L1 axis” between immune and tumor cells. CTLA-4 has a stronger affinity for B7-1 and B7-2 than CD28. CTLA-4 inhibitor (Ipilimumab) broadly enhances the immune response dependent on CD4^+^ cells. Otherwise, during ICI treatments, the “PD-1–PD-L1 axis” between immune cell and cardiomyocytes is also hindered, resulting excessive activation of immune response and cardiotoxicity. CTL-4, cytotoxic T lymphocyte-associated protein-4; TCR, T cell receptor; PD-1, programmed cell death protein-1; PDL-1, programmed cell death ligand-1.

Tumor immune escape, that is, the loss of tumor antigens, leads to a decrease in the recognition of tumor cells by the immune system. ICIs inhibit the immune escape of tumor cells, increase anti-tumor immunity, and mediate tumor regression (Figure 2). Among ICIs, PD-1 and CTLA-4 inhibitors are the most effective for tumor immunotherapy. PD-1 is a member of the CD28 superfamily. It is mainly expressed in activated T and B cells and plays an important role in maintaining the homeostasis of the immune system. It can inhibit excessive activation of T cells and exert self-protection. PD-1 is involved in inhibiting the activation of T cell receptor-proximal kinases and preventing the formation of stable T cell-APC contacts, thereby affecting signal transduction and cell-effective functions mediated by T cell antigen receptors. While tumor cells reactively upregulate the expression of PD-1 ligands (PD-L1 and PD-L2), the tumor microenvironment induces high expression of PD-1 molecules on infiltrating T cells. The pathway continues to suppress T cell activation, disabling the anti-tumor immune response, which manifests as a suppression of CD8^+^ T cells. CTLA-4 inhibits T cell activation by binding to the ligands B7-1 and B7-2. CTLA-4 has a stronger affinity for B7-1 and B7-2 than CD28. Ipilimumab (anti-CTLA-4 antibody) can broadly enhance the immune response dependent on CD4^+^ cells and was initially approved for the treatment of melanoma. ICIs that are neutralizing antibodies against CTLA-4 and PD-1 can activate the anti-tumor immune response and inhibit the growth of tumor cells by blocking the negative inhibitory effect of PD-1 and CTLA-4 on T cells. However, because the “PD-1–PD-L1 axis” and CTLA-4 pathway also mediate autoimmune damage, (Johnson et al., 2016; Varricchi et al., 2017a), ICIs can activate lymphocytes and macrophages to infiltrate nontumor as well as tumor tissues. Attacks on healthy tissues in the body result in a wide range of IRAEs (Khunger et al., 2020).

## Pathological mechanism of ICI-related cardiotoxicity

**FIGURE 3:**
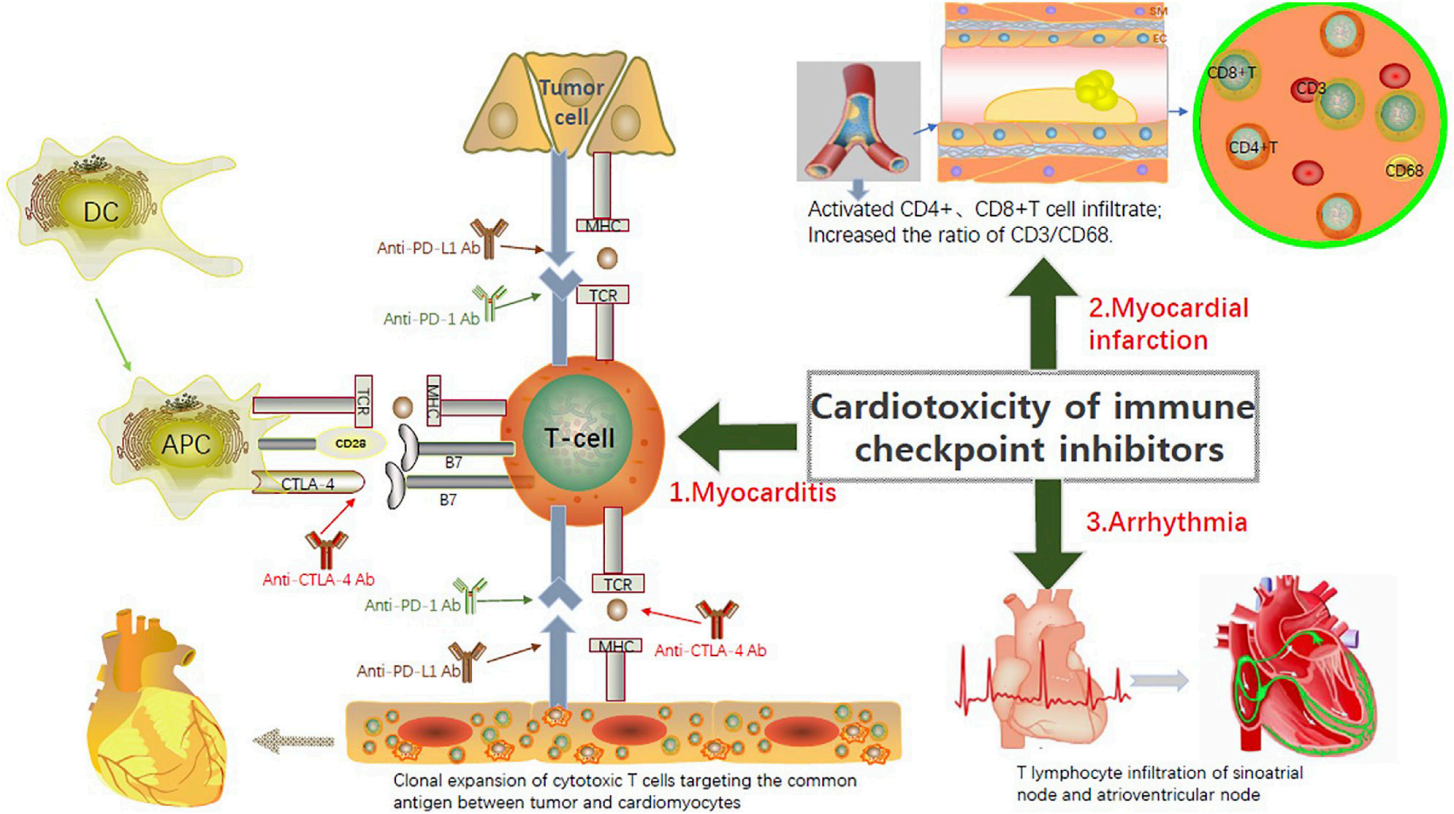
Currently recognized pathogenic mechanisms of the ICIs-related Cardiotoxicity. PD-L1 exists in cardiomyocytes and can be upregulated by a variety of cytokines, including interferon, tumor necrosis factor-α, and vascular endothelial growth factor. During ICI treatments, immunosuppressive effects mediated by the “PD-1 - PD-L1 axis” are hindered, resulting in the development of ICI-related cardiotoxicity. Although the underlying pathophysiological mechanism of ICI-related myocarditis is not totally understood, the infiltration of T cells into the injured myocardial tissue is recognized as the major pathogenic mechanism. ICI-associated inflammation and T cell activation-induced coronary vasculitis contribute to the myocardial infarction after ICI treatments. New arrhythmia is believed to be directly related to T cell-mediated cytotoxicity associated with the use of ICIs.

Although some treatments for ICI cardiotoxicity have been mastered as mentioned above, the effect is limited and it is still a barrier restricting the use of ICI drugs. Therefore, a systematic review and discussion of the possible molecular mechanisms of ICI-related cardiotoxicity are essential for identifying treatment strategies. PD-L1 is expressed in cardiomyocytes and can be upregulated by a variety of cytokines, including interferon, tumor necrosis factor-α, and vascular endothelial growth factor (Varricchi et al., 2018). Under normal circumstances, PD-L1 on the surface of cardiomyocytes binds to PD-1 on the surface of T lymphocytes to inhibit their own immune response. However, during ICI treatments, immunosuppressive effects mediated by the “PD-1 - PD-L1 axis” are hindered, resulting in the development of ICI-related cardiotoxicity (Figures 2, 3).

### Myocarditis

Although the underlying pathophysiological mechanism of ICI-related myocarditis is not totally understood, the infiltration of T cells into the injured myocardial tissue is the main reason (Salem et al., 2018). In the two cases of melanoma described byJohnson et al. (2016) patients had myositis with rhabdomyolysis, followed by massive macrophage and T cell infiltration in myocardial tissue. During the combined treatment of nivolumab and ipilimumab. Lymphocytes in the myocardium and tumor show clonality of the T cell receptor, which indicates that the heart and tumors can share the same antigen recognized by the T cell clone (Johnson et al., 2016; Varricchi et al., 2017a; Varricchi et al., 2017b). Considering the coexistence of myositis and myocarditis, skeletal muscle biopsy may be an alternative diagnostic strategy.

In a mouse myocarditis model, Lova et al. proved that CTLA-4-deficient T cells were more likely to cause substantial CD4^+^ and CD8^+^ T cell infiltration in the myocardium than CTLA-4^+^ T cells (Khunger et al., 2020; Palaskas et al., 2020). These results indicate that a lack of CTLA-4 can lead to the destruction of immune homeostasis, leading to spontaneous activation of T cells and severe myocardial damage. Similarly, animal experiments have shown that PD-L1-deficient MRL mice develop fatal lymphocytic myocarditis. Autopsy analysis revealed many macrophages, CD8^+^ and CD4^+^ T cell infiltrate in the myocardium (Khunger et al., 2020). Another animal study evaluated cynomolgus monkeys treated with ipilimumab and nivolumab. The hearts of the monkeys had CD4^+^ and CD8^+^ T cell infiltration, T cell infiltration in the monkeys was similar to that observed in humans. Immunohistochemical staining showed that PD-1 and PD-L1 were positive (Ji et al., 2019). Studies on mice have shown that genetic defects of checkpoint molecules, such as PD-1, PD-L1, and CTLA-4, increase the pathogenicity of cardiac antigen-specific effector T cells, and increase the risk of autoimmune T cell-mediated myocarditis (Grabie et al., 2019). The cardiac function of tumor-bearing mice decreased after anti-PD-1 antibody treatment. Histological examination of the left ventricle showed that it was dilated, which was related to the infiltration of CD4^+^ and CD8^+^ T cells in the myocardium. PD-1 and PD-L1 are also highly expressed in myocardial cells of rodents and humans, and their absence can cause autoimmune myocarditis (Johnson et al., 2016; Mahmood et al., 2018). The current model is that the upregulation of PD-L1 in the myocardium may be a cytokine-mediated myocardial protection mechanism, which is essential for limiting immune-mediated myocardial damage. That is, the lack of CTLA-4 and PD-1 leads to autoimmune myocarditis, suggesting that PD-1/PD-L1 and CTLA-4 play an important role in limiting T cell-mediated autoimmune myocarditis.

### Myocardial infarction

The underlying pathophysiological mechanisms of ICI-related myocardial infarction remain unclear, but three possible hypotheses have been proposed. First, the activation of ICI-related inflammation may affect atherosclerotic coronary plaques and trigger the rupture of the fibrous cap, leading to acute myocardial infarction (Chen et al., 2020). Recent single-cell sequencing and mass spectrometry analysis of human atherosclerotic plaques showed that T cells are the primary immune cell type in human atherosclerotic lesions (Johnson et al., 2016). The CD4^+^ and CD8^+^ T cells in plaques are in an activated state that promotes the formation of atherosclerotic lesions and the development of plaques to vulnerable plaques, possible causing myocardial infarction or ischemic stroke on rupturing (Johnson et al., 2016). The PD-1–PD-L1 doublet is upregulated in myocardial injury patterns (such as myocardial ischemia and myocardial infarction), and the genetic deficiency of PD-1 increases atherosclerosis in hyperlipidemic mice by increasing the function of CD4^+^ and CD8^+^ T cell effector molecules and their abundance in plaques (Seijkens et al., 2018; Herrmann, 2020). These studies have shown that immune checkpoint proteins coordinate inflammatory responses that lead to atherosclerosis. The second hypothesis is that secondary to PD-1 inhibitor (pembrolizumab) treatment, transient ST-segment elevation caused by coronary artery spasm may occur (Chen et al., 2020), but the specific mechanism is still unclear. The third hypothesis is that T cells mediate the occurrence of coronary vasculitis (Lyon et al., 2018). Further research is needed to experimentally validate these pathophysiological hypotheses. A recent study focused on determining the composition of immune cells in coronary atherosclerotic plaques in patients receiving ICI treatment, providing more insights into the pathophysiology of atherosclerosis associated with ICI treatment. It was observed that the T cells/macrophages ratio (CD3/CD68 ratio) in the plaques of patients receiving ICI treatment was significantly higher than that in the plaques of patients not receiving ICI treatment. The increase in CD3/CD68 ratio may be caused by ICIs of plaque T cell infiltration, reactivation of plaque T cells, or T cell-induced increase in macrophage apoptosis (Newman and Stone, 2019; Khunger et al., 2020).

### Arrhythmia

The cause of new arrhythmia is believed to be related directly to T cell-mediated cytotoxicity associated with the use of ICIs. Based on the histopathological findings of a patient with abnormal conduction after ICI treatment, patchy lymphocytes were found infiltrated into the sinoatrial and atrioventricular nodes (Khunger et al., 2020). In addition, we found that the risk of arrhythmia was time-dependent. Since patients with ICI-related myocarditis can develop tachyarrhythmias or bradycardia, ECG monitoring should be performed in suspected or confirmed patients.

### Myocardial injury combined with other anti-tumor drugs

Vanderburgh University Medical Center published data regarding two patients with melanoma who presented with hypertension but had no other special history of heart disease and died of fatal myocarditis 2 weeks after receiving the first dose of ipilimumab and nivolumab. The autopsy revealed a large number of T cells and macrophages that infiltrated the myocardial tissue, and the infiltrated cells were homologous to the T cells present in the tumor. Based on this information, it can be speculated that immune cells may attack normal cardiomyocytes while treating tumors and simultaneously release some cytokines, causing myocardial inflammation (Johnson et al., 2016). Therefore, combination medication is the clearest risk factor for ICI-related myocarditis, and combined conduction diseases have the highest mortality rate (Varricchi et al., 2017a; Chen et al., 2018). Immune-mediated myocarditis is characterized by fulminant progression (Han et al., 2020), and the early stage of combination medication, especially the first course of treatment, is considered a high-risk period that causes explosive myocardial damage and death. Although combination drugs have a stronger anti-tumor effect than single drugs, they are double-edged swords, and a trade-off is often faced when using combination therapy.

### Composition and function of the cardiovascular immune system

**FIGURE 4:**
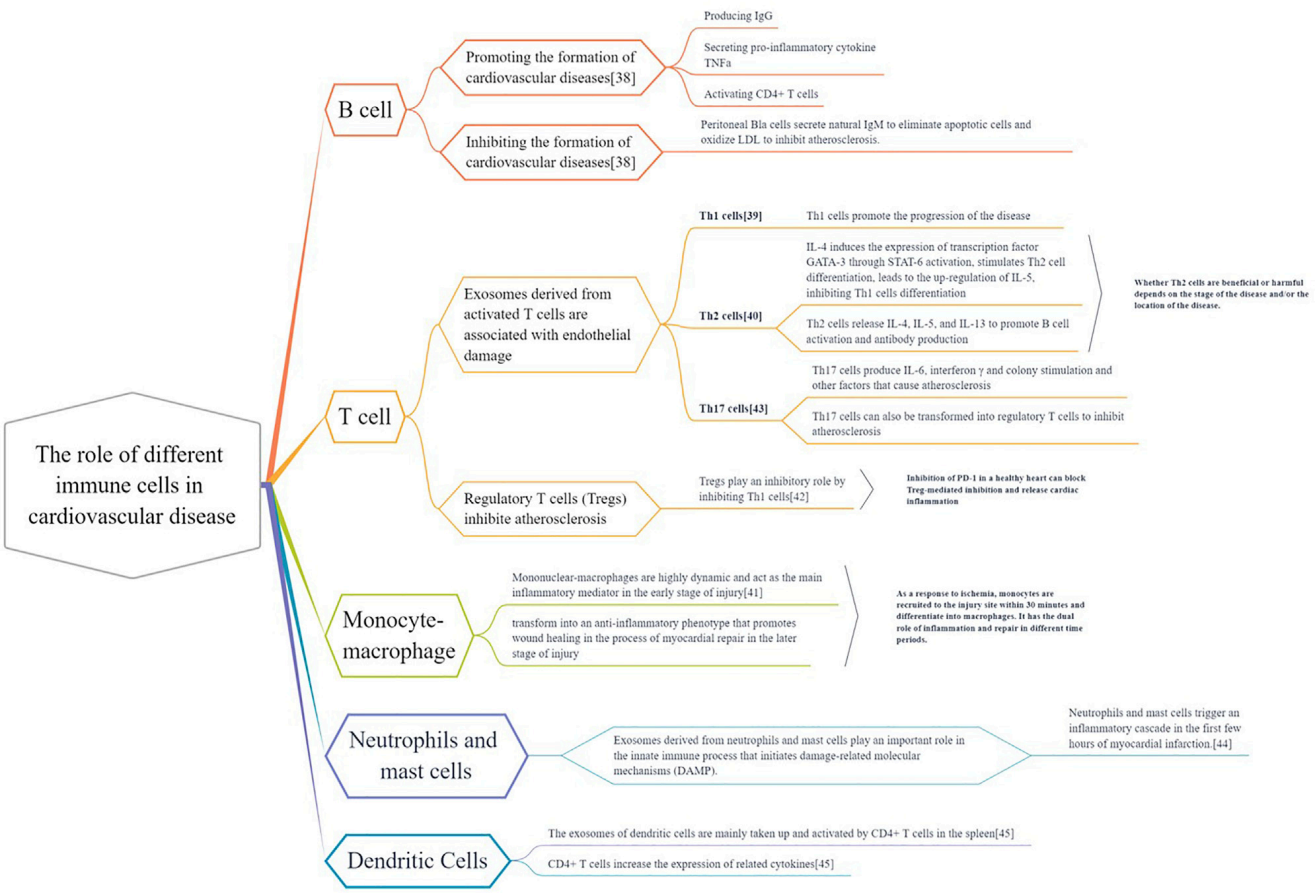
The different roles of immune cells in cardiovascular disease. Various immune cells (B cells, T cells, monocytes, macrophages, dendritic cells, neutrophils, mast cells) exist in heart and vascular system and build up cardiovascular immunologic barrier. These immune cells play multiple effects on the Cardiac and vascular parenchymal cells (cardiomyocytes, endothelial cells and smooth muscle cells) and cardiac fibroblast. They regulate the pathological process of cardiovascular diseases, contributing to or delaying the disease development.

Different immune cells (B cells, T cells, monocytes, macrophages, dendritic cells, neutrophils, mast cells) have different effects on the heart. Some cells can promote whereas some can inhibit the occurrence and development of cardiovascular diseases (Figure 4). (Gisterå and Hansson, 2017; Tabas and Lichtman, 2017; Albany et al., 2019; Wu et al., 2019; Martini et al., 2020; Saigusa et al., 2020; Varricchi et al., 2020; Ferrari et al., 2021)

## Current treatment strategy for ICI-related cardiotoxicity

According to the severity of the condition, ICI-related cardiotoxicity is divided into four levels: 1) level 1, which includes asymptomatic with laboratory abnormalities (such as abnormal detection of cardiac biomarkers and abnormal electrocardiogram); 2) level 2, which includes mild symptoms with screening test; 3) level 3, which includes moderately abnormal or mildly active symptoms; and 4) level 4, which includes moderate to severe, even life-threatening, decompensated heart damage requiring abnormal intravenous medication or interventional therapy. (Liu and Wu, 2020). The first step in dealing with immunomodulator-related heart damage is to determine and evaluate the severity of cardiotoxicity. Because patients have different risks of ICI cardiotoxicity, a personalized clinical approach is crucial. An in-depth baseline assessment should be carried out for all patients, and monitoring strategies for high-risk patients should be considered too quickly detect immune-related toxicity and side effects. In a previous study, we focused on the first 12 weeks when the risk of cardiotoxicity was higher. However, whether a previous history of myocardial damage (such as myocardial infarction or myocarditis) is a risk factor for ICI-related cardiotoxicity remains unclear. Patients with a known history of autoimmune diseases, such as rheumatoid arthritis, systemic lupus erythematosus, and sarcoidosis, maybe at a higher risk, especially those with previous heart involvement. Therefore, it is recommended that in addition to the oncologist, a cardiologist is consulted to detect the presence of any cardiovascular severities such that the patient is provided with the best treatment plan (Konala et al., 2020; Romitan et al., 2020). The treatment strategy for ICI-related cardiovascular complications is divided into the following three aspects: stop using ICIs to prevent further toxicity, start supportive treatment for cardiac complications, and use immunosuppression agents to suppress excessive inflammation.

Due to the long functional half-life of ICIs, terminating ICI treatment may not immediately reverse its cardiotoxicity. The decision to stop ICI treatment requires a thorough discussion between an oncologist and a cardiovascular expert. Current guidelines recommend ending ICI treatment when patients develop grade 3 or 4 ICI-related toxicities. However, stopping ICI treatment for patients with asymptomatic abnormalities in the laboratory (level 1) or abnormal detection and mild symptoms (level 2) remains controversial. Some studies reported that after the test is normalized, for patients with grade 1 or 2 ICIs-related toxicity, ICI treatment can be considered again, but ICI treatment should be permanently discontinued in case of grade 3 and 4 toxicity. According to the latest guidelines of ASCO, patients with any ICI-related toxicity greater than grade 1 (asymptomatic biomarker elevation) should permanently stop ICI treatment (Stein-Merlob et al., 2021).

If necessary, patients with ICI-related heart injuries can receive effective supportive treatment. For example, patients with heart failure should receive medications recommended by the guidelines, including β-blockers and ACEI/ARB. Patients with life-threatening tachyarrhythmia can use appropriate antiarrhythmic drugs, such as intravenous amiodarone. According to the needs of advanced conductive disease, temporarily or possibly permanently placing a pacemaker should be considered. (Yang and Asnani, 2018). If the patient has persistent refractory heart failure, in addition to vasoactive drugs, intra-aortic balloon counter pulsation or extracorporeal membrane oxygenation supportive therapy should be given.

Immunosuppressive therapy can also be applied to overactive T cell responses. Corticosteroids are currently considered as the first-line immunosuppressive drug for ICI-related myocarditis. (Yang and Asnani, 2018; Romitan et al., 2020; Stein-Merlob et al., 2021; Waliany et al., 2021).However, many studies have shown that corticosteroids alone may not be enough to improve immune-mediated adverse cardiac reactions. During steroid therapy, patients with ICI-related cardiac events may progress to malignant arrhythmias and severe heart failure symptoms. Patients with poor corticosteroid response should use other immunosuppressive drugs, including immunoglobulin, mycophenolate mofetil, tacrolimus, and infliximab, as well as other second-line treatment options. In severe cases, plasma exchange can be performed. (Asnani, 2018; Chen et al., 2018; Yang and Asnani, 2018; Zhou et al., 2019; Liu and Wu, 2020).

## Perspective

ICIs has a significant anti-tumor effect, which brings good news for the treatment of many refractory or advanced tumors. As ICI treatment has expanded to encompass a variety of cancer types, and more novel ICIs are entering clinical trials, more patients receive the benefit from the novel immunotherapy. But they will also experience serious irAEs, which usually force them to abandon treatment or lead to death. Actually, ICI-mediated cardiotoxicity is relatively rare, but when it does occur clinically, it is often serious and potentially fatal. Current treatment strategies still struggle to correct heart injury and cardiac dysfunction. Most deaths have been reported so far. Meanwhile, ICI-related cardiotoxicity also limits the participation of patients with underlying cardiovascular diseases in clinical trials of ICIs, which tremendously narrows the application. At present, the pathological mechanism of cardiotoxicity after ICI treatments is unclear, and the explanation of cardiotoxicity induced by excessive activation of T cells is still incomplete and controversial, which thus greatly restricts the modification of clinical treatment plans and the development of novel therapy. Therefore, it is inevitably urgent to understand ICI-related cardiovascular side effects, predisposing risk factors, and the underlying pathological mechanisms. Moreover, it is indeed important to monitor early toxicity and establish appropriate treatment for patients with ICI-related cardiotoxicity. Close collaboration between cardiologists and oncologists is needed to improve the outcomes and survival of patients with cancer receiving ICIs. Additionally, prospective studies aiming to record and characterize cardiac function during ICI therapy are necessary, and systematic registration of patients with ICI-related cardiotoxicity will help to develop standardized treatment recommendations for this collective.
