# Supplementary material for: Cardiotoxicity associated with immune checkpoint inhibitors: Current status and future challenges
Source: Front Pharmacol. 2022 Aug 30;13:962596. doi: 10.3389/fphar.2022.962596 (PMC9468595; doi:10.3389/fphar.2022.962596)
Supplement: Supplementary file 1 [file DataSheet1.PDF]

Table S1. The immune-related myocarditis induced by ICI treatments in clinical trials.

ICIs		Tumor types	Clinical trial	irAEs				References
				> grade 3	Myocarditis		Others cardiotoxicity	
					Morbidity	Outcome		
**PD-1 antibody**	Pembrolizumab	Advanced urothelial cancer	Phase 2<br>multi-center	58/370 (15%)	Grade 4, 1/370 (<1%)<br>Grade 5, 1/370 (<1%)	died	/	ClinicalTrials.gov (NCT02335424)<br>Arjun V Balar<br>PMID: 28967485
		Thymic carcinoma	Phase 2<br>single-center	6/40<br>(15%)	Grade 4, 2/40 (5%)	Recovered	/	ClinicalTrials.gov (NCT02364076)<br>Giuseppe Giaccone<br>PMID: 29395863
		Thymoma;<br>Thymic carcinoma	Phase 2<br>single-center	Thymoma:5/7 (71.4%)<br>Thymic carcinoma:4/26 (15.4%)	Thymoma: Grade 4, 3/7 (42.9%)<br>Total: 3/33 (9%)	Recovered	/	Jinhyun Cho<br>PMID: 29567210
	Pembrolizumab + pomalidomide and dexamethasone	Relapsed or refractory multiple myeloma	Phase 3<br>multi-center	75/120 (63%)	Grade 5, 1/120 (0.8%)	died	Cardiac failure, myocardial infarction, pericardial hemorrhage	ClinicalTrials.gov (NCT02576977)<br>Maria-Victoria Mateos<br>PMID: 31327687

Pembrolizumab + lenalidomide and dexamethasone	Treatment-naive multiple myeloma	Phase 3 multi-center	81 (54%)	Grade 5, 1/149 (0.7%)	died	Cardiac arrest, cardiac failure, cardiorespiratory arrest, Myocardial infarction	ClinicalTrials.gov (NCT02579863) Saad Zafar Usmani PMID: 31327689
Pembrolizumab + p53MVA vaccine	Advanced solid cancers	/	/	Grade 5, 1/11 (9%)	died	/	ClinicalTrials.gov (NCT02432963) V Chung PMID: 30094792
Camrelizumab	Advanced or metastatic oesophageal squamous cell carcinoma	Phase 3 multi-center	44/228 (19%)	Grade 5, 1/228 (<1%)	died	/	ClinicalTrials.gov (NCT03099382) Jing Huang PMID: 32416073
Nivolumab	biliary tract cancer	Phase 1 multi-center	Monotherapy cohort: 3/30 (10%); Combined therapy cohort: Grade 3, 27/30 (90%), Grade 4, 16/30 (53%)	Combined therapy cohort: Grade 3, 1/30 (3%)	/	/	clinicaltrials.jp (JapicCTI-153098) Makoto Ueno PMID: 31109808

**PD-L1 antibody**	Avelumab	Advanced non-small-cell lung cancer	Phase 3 multi-center	39/393 (10%)	Grade 5, 1/393 (<1%)	died	Acute cardiac failure, Grade 5, 1/393 (<1%) dead	ClinicalTrials.gov (NCT02395172) Fabrice Barlesi PMID: 30262187
	Avelumab+ axitinib	Advanced clear-cell renal-cell carcinoma	Phase 1b multi-center	32/55 (58%)	Grade 5, 1/55 (2%)	died	/	ClinicalTrials.gov (NCT02493751) Toni K Choueiri PMID: 29530667
	Durvalumab	Advanced solid tumors	Phase 1b multi-center	Durvalumab: 28/136 (21%); Durvalumab+tremelimumab: 27/112 (24%);	Etoposide/Platinum Cohorts: Grade 5 1/22 (5%)	died	/	clinicaltrials.gov (NCT02537418) PMID: 32169783 Rosalyn A Juergens

**Combination (Anti-PD-1/PD-L1+ CTLA-4 antibody)**	Pembrolizumab + ipilimumab	Advanced Melanoma	Phase 2 multi-center	PEM200+IPI50: 12/51(24%); PEM200+IPI100: 20/51(39%)	PEM200+IPI50: Grade 5, 1/51(2%)	died	PEM200+IPI50: Atrioventricular block, Grade 3, 1/51(2%)	ClinicalTrials.gov (NCT02089685) Georgina V Long PMID: 34210681
	Pembrolizumab Nivolumab Ipilimumab Tremelimumab Atezolizumab Avelumab Durvalumab Or Combination	Head and neck Breast Hodgkin's lymphoma Melanoma Non-small cell lung cancer Small cell lung cancer Pancreatic Renal cell carcinoma Glioblastoma other	multi-center	/	Total: 1.14% Monotherapy: 66%	/	Abnormal ECG, elevated troponin, acute CV symptoms	Syed S Mahmood PMID: 29567210
	Pembrolizumab Nivolumab Ipilimumab Or Combination	Metastatic Merkel cell carcinoma or melanoma with neuromuscular disorders	multi-center	49%	10/38 (26.3%)	1/10 (10%) died	/	Alvaro Moreira PMID: 30453170

	Nivolumb+ Ipilimumab	Melanoma Metastatic to the Brain	Phase 2 multi-center	56/94 (60%)	Grade 5, 1/94 (1%)	died	/	ClinicalTrials.gov (NCT02320058) Hussein A Tawbi PMID: 30134131
	Nivolumb+ Ipilimumab	Melanoma with at least one non-irradiated brain metastasis measuring	Phase 2 multi-center	56/101 (55%)	Grade 5, 1/101 (1%)	died	/	ClinicalTrials.gov (NCT02320058) Hussein A Tawbi PMID: 34774225
	Nivolumab Pembrolizumab Durvalumab Nivolumab+ipilimumab	Metastatic Melanoma, NSCLC, Breast	/	4/10	4/10 (40%)		elevated creatine kinase levels	Mehdi Touat PMID: 30089619
	Anti-PD-1/PD-L1 Ipilimumab Combination	Melanoma Lung cancer others	Vigilyze database	Fatal irAEs 613/31059 (2%)	Fatal Myocarditis Ipilimumab: 3/193 (2%) Anti-PD-1/PD-L1 27/333 (8%) Combination: 22/87 (25%)	39.7% died	/	Daniel Y Wang PMID: 30242316
	Nivolumab or Nivolumb+ Ipilimumab	/	Bristol-Meyers Squibb database	/	Nivolumab: 1/2000 (<1%) Combination: 1/300 (0.3%)	/	/	Hassan Mir PMID: 29980467
